# The Anti-Porcine Parvovirus Activity of Nanometer Propolis Flavone and Propolis Flavone *In Vitro* and *In Vivo*

**DOI:** 10.1155/2015/472876

**Published:** 2015-02-26

**Authors:** Xia Ma, Zhenhuan Guo, Zhiqiang Shen, Yonglu Liu, Jinliang Wang, Yunpeng Fan

**Affiliations:** ^1^Medicinal Engineering Department of Henan University of Animal Husbandry and Economy, Zhengzhou, Henan 450011, China; ^2^Binzhou Animal Science & Veterinary Medicine Academy of Shandong Province, Binzhou, Shandong 256600, China; ^3^College of Veterinary Medicine, Northwest A&F University, Yangling, Shaanxi 712100, China

## Abstract

*Objectives*. The present study was conducted to evaluate the activity of nanometer propolis flavone (NPF) on inhibiting porcine parvovirus (PPV) *in vitro* and *in vivo*. *Methods*. *In vitro*, the effect of NPF on cellular infectivity of PPV was carried out before and after adding drug and simultaneous adding and PPV after being mixed. *In vivo*, the anti-PPV effect of NPF in guinea pigs was performed. *Results*. The results showed that NPF could significantly inhibit PPV infecting porcine kidney- (PK-) 15 cells compared with propolis flavone (PF), and the activity of NPF was the best in preadding drug pattern. NPF at high and medium doses was able to observably restrain PPV copying in lung, gonad, blood, and spleen, decrease the impact of PPV on weight of guinea pigs, and improve hemagglutination inhibition (HI) of PPV in serum. In addition, it could also increase the contents of IL-2 and IL-6 in serum after PPV challenge. *Conclusion*. These results indicated that NPF could significantly improve the anti-PPV activity of PF, and its high concentration possessed the best efficacy. Therefore, NPF would be expected to be exploited into a new-style antiviral drug.

## 1. Introduction

In recent years, the animal infectious diseases, especially viral diseases, such as severe acute respiratory syndrome, untypical classical swine fever, swine hyperpyrexia disease, Newcastle Disease, and cow transmissible spongiform encephalopathy, are worldwide of concern as they take a stage of comeback with spread quickly and emerge continuously and constantly cause an enormous loss in domestic animal [1]. Porcine parvovirus (PPV) characterized as a member of the autonomous parvoviruses is one of main pathogens that could cause reproductive failure in pregnant sows, which is characterized by fetal and embryonic death, stillbirths, mummification, and delayed return to oestrus [2]. Although the acute infection of postnatal and nonpregnant pigs is often subclinical, PPV has been linked to interstitial nephritis in slaughter aged pigs [3], skin lesion occurrence in piglets [4], and nonsuppurative myocarditis in lactating piglets. Recently, PPV has gained attention as an agent able to potentiate the effects of porcine circovirus type 2 (PCV2) infection in the clinical course on postweaning multisystemic wasting syndrome, a worldwide significant economical disease [5]. Besides its association with the pathological conditions and abovementioned clinical, vaccination with inactivated PPV is generally considered to offer good protection against PPV and is used to control reproductive failure due to PPV. Several inactivated aluminum hydroxide adjuvant vaccines for PPV immunoprophylaxis are available in the United States and oil emulsion adjuvant vaccine of PPV is licensed by veterinary drug standard of the People's Republic of China.

For the past few years, it has been demonstrated that many Chinese herbal medicines (CHMs) or their ingredients (CHMIs) possess antiviral effect* in vitro* and* in vivo* [6]. Some CHMs or CHMIs such as isatidis radix, angelica polysaccharides, and astragalus polysaccharides which were isolated from* Isatis indigotica* Fort.,* Angelica sinensis* (Olive.) Diels, and* Astragalus membranaceus* (Fisch.), respectively, have been developed into antiviral or antibacterial agents [7]. On the one hand, many researches demonstrated that CHMs or CHMIs contain many antiviral active materials, but many active materials had still not been discovered. On the other hand, CHMs or CHMIs possess many biological activities, such as inhibiting viral processes (adsorption, transcription, replication, and release), adjusting immunologic function, and antioxidant, which shows dependable biological functions with light side effects and little toxicity [8]. Moreover, new virus or variant strains of old virus arise continually. So, antiviral drug screened from CHMs became a hotspot for many investigators.

Propolis, applied empirically as a traditional cure in folk medicine for centuries, is a mixture formed by honeybees with various plants resinous and their mandibular gland secretion. Propolis is rich in biochemical constituents, and more than 300 compounds have been identified in it. Flavone is one of the most important pharmacologically active constituents in propolis and is thought to be responsible for many of its biological and pharmacological activities. It is well known for valuable health benefit and is reported to possess reliably biological activities, including antivirus, immune enhancement, antioxidation, antibiosis, hepatoprotection, anticancer, and antifatigue [9–11]. It is an excellent immune system booster and natural antibiotic with no side effects. Recently, propolis has become an issue of increasing interest among the investigators owing to its versatile biological activities. Due to these biological activities, propolis has been broadly marketed as the health-food and alternative medicine in various parts of the world. Propolis flavone (PF), a kind of ingredient extracted from propolis, as a harmless natural adjuvant and antivirus has been used in chickens vaccinated with activated or inactivated vaccine. Many studies all proved that PF could improve the immune-enhancing activity in the cellular and humoral immune response [9]. In addition, our previous study also demonstrated that the adjuvant effects and feature of PF on inactivated PPV vaccine to guinea pigs had been considered successfully in cellular and humoral immunity [12].

Our previous researches showed that PF possessed a better immune enhancement and anti-PPV activity. In the present study, PF was processed to nanometer PF (NPF) by nanotechnology. Besides, the authors determined the effects of NPF on anti-PPV* in vitro* and* in vivo*. The aim of this study was to assess the antiviral effect and characteristic of NPF compared with PF on PK-15 cell and guinea pigs, respectively, and afford theoretical evidence for NPF developing into a new antiviral drug.

## 2. Materials and Methods

### 2.1. Materials

PF was kindly provided by Binzhou Animal Science and Veterinary Medicine Academy of Shandong Province. Ethyl acetate, twain-80, and span-80 were purchased from BASF (Berlin, Germany). PPV (TCID_50_: 10^−5^) and PK-15 cell were supplied by China Institute of Veterinary Drug Control. DMEM (GIBCO) with the supplement of 100 IU mL^−1^ streptomycin, 100 IU mL^−1^ benzylpenicillin, and 10% fetal bovine serum was used for resuspending and washing cells, culturing the cells, and diluting mitogen. The 3-(4,5-dimethylthiazol-2-yl)-2,5-diphenyltetrazolium bromide (MTT, American Co.) was dissolved into 5 mg·mL^−1^ with calcium and magnesium-free phosphate-buffered saline (PBS, pH 7.2). Dimethyl sulfoxide was produced by Zhengxing Chemical Co. Ltd. (Suzhou, China). Dimethyl sulfoxide (DMSO) was the product from Chemical Reagent Ltd of Shanghai Lingfeng. These reagents were filtered through a 0.22 *μ*m millipore membrane filter. The enzyme-linked immunosorbent assay (ELISA) kits for IL-2, IL-6, and *γ*-IFN were purchased from R&D Systems Inc., Minneapolis, USA. Ethyl acetate and ethanol were of analytical grade and supplied from Kermel Chemical Co. Ltd. (Tianjin, China). All other chemicals are of analytical grade.

### 2.2. Preparation of Nanometer Propolis Flavone

Based on the consequences of preliminary experiments, NPF were prepared at desired component ratios. Therefore, ethyl acetate, ethanol, and deionized water were selected as the oily phase, surfactant, cosurfactant, and aqueous phase, respectively. Besides, the optimal ratio of surfactant and cosurfactant was installed as 3 : 2 (w/w) by twain-80 and span-80. First, PF was mixed into the compounds of oil, surfactant, and cosurfactant with certain ratios. Then water was added into the compounds dropwise and agitating for 24 h at 25°C. And then the solution was filtered by 0.45 *μ*m membrane. The average particle size and zeta potential of NPF were 35.5 nm and −4.12 mV. Under electron microscope, NPF had a closely spherical appearance. The diameters of the NPF determined by transmission electron microscopy were in ideal agreement with the particle sizes determined [13].

### 2.3. The Design of Experiment* In Vitro*

#### 2.3.1. Determination of PK-15 Safe Concentration

When PK-15 grew into monolayer in 96-well plates, the series of concentrations NPF and PF at 2000, 1000, 500, 250, 125, 62.5, 31.2, 15.6, 7.8, and 3.9 *μ*g·mL^−1^ were added into the plates, six wells each concentration. After a culture for 68 h at 37.5°C in a humid atmosphere of 5% CO_2_, 20 *μ*L MTT was added into each well and sequentially incubated for 4 h; the supernatant was removed and then 100 *μ*L of DMSO was added into. The plates were shaken for 5 min to dissolve the DMSO crystals completely. Each well of absorbance at 570 nm (*A*_570_ value) was determined by micro liter enzyme-linked immunosorbent assay reader (Type: DG-3022, Vacuum Tube Manufacturer of East China).

The value of *A*_570_ is correlation to the live cells number; the bigger *A*_570_ value is, the more live cells are. When *A*_570_ values of NPF and PF groups were not significantly lower than those of cells control groups, which indicated that NPF and PF had no cytotoxicity, then the corresponding concentrations were considered as maximal safety concentration for PK-15.

#### 2.3.2. Antiviral Assays

According to the results of safety concentration, five NPF and PF concentrations, from 250 to 3.9 *μ*g·mL^−1^, were selected for measurement of antiviral activity by MTT method and real-time fluorescence quantitative (RTFQ) PCR. When PK-15 cell cultured into monolayer, five NPF and PF dilutions at 250, 125, 62.5, 31.2, and 15.6 *μ*g·mL^−1^ and PPV were added into cell plate, respectively, in three patterns.

*Before Adding NPF and PF.* Firstly NPF or PF solutions were added into PK-15 cell plate, 100 *μ*L/well, and six wells per concentration. After being incubated for 2 h at 37.5°C in 5% CO_2_, NPF or PF solutions were removed; the cells were washed twice with Hanks' solution and then the PPV solution was added.

*After Adding NPF and PF.* Firstly PPV solution was added into PK-15 cell plate. After being incubated for 2 h, PPV solution was removed, the cells were washed twice with Hanks' solution and then NPF or PF solutions were added, six wells for each concentration.

*Mixed Adding of NPF or PF with PPV.* The NPF or PF solutions at each concentration were mixed with PPV solution and incubated for 4 h at 4°C and then added into PK-15 cell plate, six wells for each concentration.

All PK-15 cell plates were placed into 5% CO_2_ incubator at 37.5°C. When the PPV control groups showed markedly cytopathic effect (CPE) after 72 h, the PK-15 cell viability was determined by the MTT assay. The PPV content in PK-15 cell was determined with RTFQ PCR. The mean cellular values of *A*_570_ and virus content were used as the indicator of antiviral activity. When the *A*_570_ value of NPF or PF group was significantly higher than that of PPV control group and the virus content of NPF or PF group was significantly lower than that of PPV control group, it indicated that the corresponding NPF or PF had significant antiviral activity [14].

### 2.4. The Design of Experiment* In Vivo*

#### 2.4.1. Experimental Animals

Britain White guinea pigs (Four-month-old, equal male and female) were purchased from Experimental Animal Center of Chinese Medicine (Beijing, China). The guinea pigs were raised in 80 cm × 50 cm × 40 cm wire cages, 5 each cage, in air-conditioned rooms at 18–21°C, and illuminated for 24 h receiving water and feeding* ad libitum*. And hemagglutination inhibition (HI) antibody against PPV of guinea pigs is zero before the experiment.

#### 2.4.2. Antiviral Experiment

320 guinea pigs were divided into 8 groups randomly; the guinea pigs in six drug groups were intragastric administration, respectively, with high (8 g/kg·d), middle (4 g/kg·d), and low (2 g/kg·d) dose of NPF and PF, in challenge control (CC) and blank control (BC) group, with the dose of physiological saline, for continuous three days. On day of intragastric administration, the guinea pigs in six groups and CC group were challenged with 0.5 mL of PPV, and the challenged dosage was 4.5 log_2_ titers of median tissue culture infective dose (TCID_50_) mL^−1^. On days 3, 7, 14, and 21 after challenge the blood was sampled for HI serum antibody titers analysis by micromethod and the contents of IL-2, *γ*-IFN, and IL-6 by ELISA. The PPV contents in tissue of lungs, gonad, spleen, liver, and blood were measured by method of RTFQ PCR. And the weight of guinea pigs in each groups was also observed on day 21 after challenge.

#### 2.4.3. HI Antibody Titre of PPV Determination

Blood samples (0.5 mL per guinea pig) from ear vein were drawn into Eppendorf tubes and placed to clot at 37°C for 0.5 h. Serum was separated for measurement of HI antibody by centrifugation. Briefly, twofold serial dilution of 50 *μ*L serum was added into a 96-well V-shaped bottom microtiter plate containing 50 *μ*L CMF-PBS in all wells after being inactivated at 56°C for 30 min; then 50 *μ*L PPV antigen (4 HA units) was joined into all the wells except for the last row served as controls. Serum dilutions ranged from 1 : 2 to 1 : 2048 and were joined into 96-well V-shaped bottom microtiter plate. The serum-antigen mixture was incubated at 37°C for 10 min. 50 microlitres of 1% rooster erythrocytes suspension was joined into each well and reincubated at 37°C for 30 min. A negative serum, a positive serum, antigens, and erythrocytes were also included. The highest serum dilution causing completely inhibition was regarded as the endpoint. The geometric mean titer was marked as reciprocal log_2_ values of the highest HI antibody titer dilution.

#### 2.4.4. Cytokine Determination

Serum concentrations of cytokines (including IL-2, *γ*-IFN, and IL-6) were determined by ELISA according to instructions of manufacturer. Briefly, the capture antibody specific for each cytokine was coated into a 96-well flat bottom microtiter plate (Nunc, USA). 10 *μ*L of the serially diluted specific standards and serum was added to the respective wells, and then the plate was washed and blocked. Then the captured cytokine was detected by means of the specific conjugated detection antibody before a series of washing. The substrate reagent was attended into each well after color change and the plate was counted at 450 nm using an ELISA reader (BIO-TEK. Instruments INC). Cytokine concentrations were accomplished by a standard curve plotting* A*_450_ value against each dilution of standards concentration.

#### 2.4.5. PPV Content Determination

Viral genomic DNA was extracted from the tissue of challenged guinea pigs using RNA/DNA Extraction Kit Ver. 3.0 (TaKaRa, Dalian, China) according to the protocol of manufacturer. A series of tests was performed to optimize the RTFR PCR protocol, including PCR cycling parameters and reagent concentration. Optimization of each singleplex real-time PCR assay of PPV was essential for the subsequent PCR reaction. Water instead of template DNA used as negative controls using was run with each test. All RTFR PCR reactions were performed in triplicate. A total of tissue and serum samples were tested for PPV by RTFR PCR and conventional PCR assays. The conventional RTFR PCR assays conditions were the same as the standard plasmid template preparation described [15].

#### 2.4.6. Statistical Analysis

Data are shown as the mean ± S.D. LSD and Duncan's multiple range test was used to compare the differences among groups. Significant difference was considered at *P* < 0.05.

## 3. Result

### 3.1. Experiment* In Vitro*

#### 3.1.1. Cytotoxicity

The PK-15 cell *A*_570_ values of every NPF and PF groups were listed in Table 1. In NPF group, the PK-15 cell *A*_570_ values of 250–3.9 *μ*g·mL^−1^ groups were not observably lower than that of cell control group, so its maximal safety concentration could be ascertained as 250 *μ*g·mL^−1^. In NP group, the PK-15 cell *A*_570_ values of 500–3.9 *μ*g·mL^−1^ groups were not significantly lower than that of cell control group, and its maximal safety concentration could be determined as 500 *μ*g·mL^−1^. In order to observe their action at the same level, the NPF and PF maximal safety concentration were supposed as 250 *μ*g·mL^−1^.

#### 3.1.2. The Cytoactivity of PK-15 Cell Challenged with PPV

**Figure 1:**
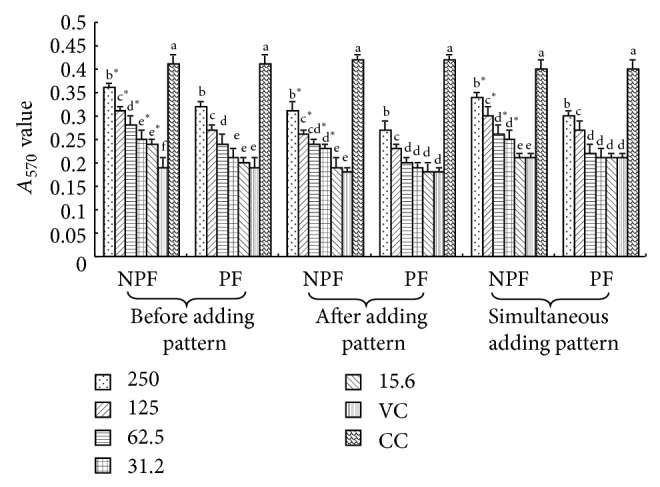
*A*_570_ values of every group in 3 adding drug patterns (*A*_570_).  ^a–f^Bars in the same mode marked without the same superscripts differ significantly and  ^*^ in the same pattern differ significantly between NPF and PF (*P* < 0.05). NPF, nanometer propolis flavone; PF, propolis flavone; VC, virus control; CC, Cell control.

The cytoactivities of PK-15 cell challenged with PPV are shown in Figure 1. In preadding pattern, the PK-15 cell *A*_570_ values of NPF at 250–15.6 *μ*g·mL^−1^ concentration groups were observably larger than those of the PPV control (*P* < 0.05). The PK-15 cell *A*_570_ values of NP at 62.5, 125, and 250 *μ*g·mL^−1^ groups were significantly larger than those of the PPV control (*P* < 0.05). And the PK-15 cell *A*_570_ values of NPF at 250–31.2 *μ*g·mL^−1^ groups were significantly larger than those of PF (*P* < 0.05). In postadding pattern and in simultaneous adding pattern, the PK-15 cell *A*_570_ values of NPF at 250–31.2 *μ*g·mL^−1^ concentration groups were observably larger than those of the PPV control (*P* < 0.05). The PK-15 cell *A*_570_ values of NP at 125 and 250 *μ*g·mL^−1^ groups were significantly larger than those of the PPV control (*P* < 0.05). And the PK-15 cell *A*_570_ values of NPF at 250–31.2 *μ*g·mL^−1^ groups were observably larger than those of PF (*P* < 0.05).

#### 3.1.3. The PPV Content in PK-15 Cell after Challenge

**Figure 2:**
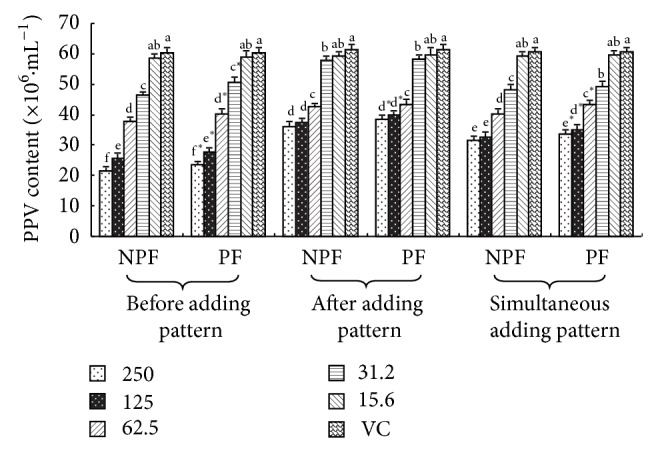
PPV content of every group in 3 adding drug patterns (×10^6^·mL^−1^).  ^a–f^Bars in the same mode marked without the same superscripts differ significantly and  ^*^ in the same pattern differ significantly between NPF and PF (*P* < 0.05). NPF, nanometer propolis flavone; PF, propolis flavone; VC, virus control.

The PPV content in PK-15 cell after challenge is illustrated in Figure 2. In preadding pattern, at 250–31.2 *μ*g·mL^−1^ concentration the PPV contents in PK-15 cell of NPF and PF groups were significantly lower than those of the PPV control, and NPF groups were observably lower than those of PF groups (*P* < 0.05). In postadding pattern, the PPV contents in PK-15 cell of NPF and PF at 250–31.2 *μ*g·mL^−1^ concentration groups were markedly lower than those of the PPV control, and at 250–125 *μ*g·mL^−1^ NPF groups were observably lower than those of the PF groups (*P* < 0.05). In simultaneous adding pattern, the PPV contents in PK-15 cell of NPF and PF at 250–31.2 *μ*g·mL^−1^ concentration groups were significantly lower than those of the PPV control, and at 250–62.5 *μ*g·mL^−1^ NPF groups were observably lower than those of the PF groups (*P* < 0.05).

### 3.2. Experiment* In Vivo*

#### 3.2.1. The Dynamic Changes of PPV Content in Lung, Gonad, and Blood

**Figure 3:**
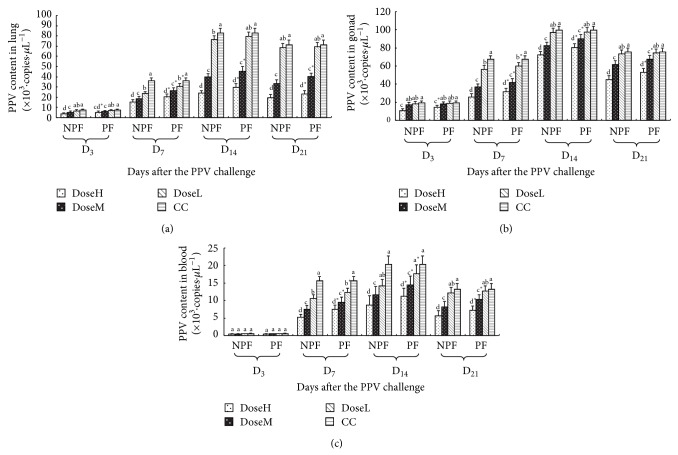
The dynamic changes of PPV content of every group in lung, gonad, and blood (×10^3^·copies·*μ*L^−1^).  ^a–d^Bars in the same mode marked without the same superscripts differ significantly and  ^*^ in the same time differ significantly between NPF and PF (*P* < 0.05). NPF, nanometer propolis flavone; PF, propolis flavone; DoseH, high dose; DoseM, middle dose; DoseL, low dose; CC, challenged control. (a) PPV content of every group in lung; (b) PPV content of every group in gonad; (c) PPV content of every group blood.

The dynamic changes of PPV content of every group in lung, gonad, and blood are shown in Figures 3(a), 3(b), and 3(c), respectively. On day 7 after challenge, the PPV contents in CC groups of lung, gonad, and blood were the highest and observably higher than those in NPF and PF groups (*P* < 0.05). On days 14 and 21 after challenge, the lung, gonad, and blood PPV contents in NPF and PF groups were significantly lower than those in CC groups at high and middle dose (*P* < 0.05). And at high and middle dose, the lung, gonad and blood PPV contents in NPF groups were markedly lower than those in PF groups (*P* < 0.05).

#### 3.2.2. The Dynamic Changes of PPV Content in Spleen and Liver

**Figure 4:**
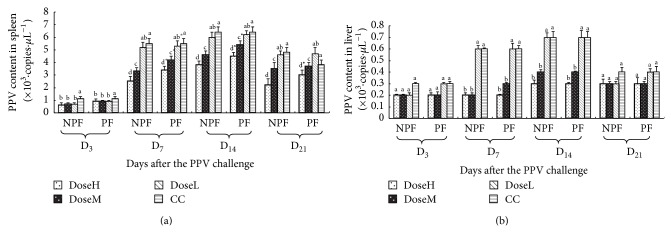
The dynamic changes of PPV content of every group in spleen and liver (×10^3^·copies·*μ*L^−1^).  ^a–d^Bars in the same mode marked without the same superscripts differ significantly and  ^*^ in the same time differ significantly between NPF and PF (*P* < 0.05). NPF, nanometer propolis flavone; PF, propolis flavone; DoseH, high dose; DoseM, middle dose; DoseL, low dose; CC, challenged control. (a) PPV content of every group in spleen; (b) PPV content of every group in liver.

The dynamic changes of PPV content of every group in spleen and liver are illustrated in Figures 4(a) and 4(b), respectively. On days 3, 7, 14, and 21 after challenge, the spleen PPV contents in NPF and PF groups were observably lower than those in CC groups at high and middle dose (*P* < 0.05). And on days 7, 14, and 21, the spleen PPV contents in NPF were significantly lower than those in PF groups at high and middle dose (*P* < 0.05). The PPV content in liver was low from 3 to 21 after challenge, and on days 7 and 14, the liver PPV contents in NPF and PF groups were significantly lower than those in CC groups at high and middle dose (*P* < 0.05).

#### 3.2.3. The Variation of HI Antibody Titer

**Figure 5:**
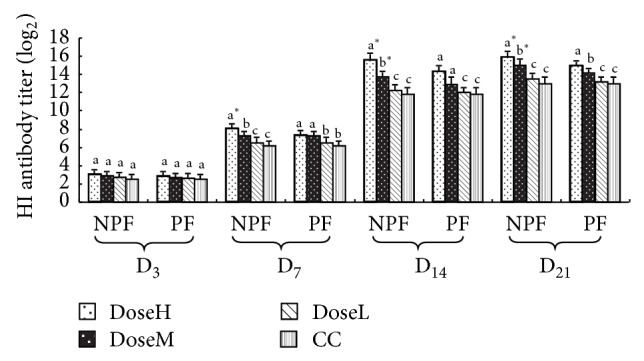
The variation of HI antibody titer in every group after challenge (log_2_).  ^a–c^Bars in the same mode marked without the same superscripts differ significantly and  ^*^ in the same time differ significantly between NPF and PF (*P* < 0.05). NPF, nanometer propolis flavone; PF, propolis flavone; DoseH, high dose; DoseM, middle dose; DoseL, low dose; CC, challenged control.

The results are shown in Figure 5. The HI antibody titers of PPV in NPF and PF groups were significantly lower than those in CC groups at high and middle dose on days 7, 14, and 21 after challenge (*P* < 0.05). And on day 7, HI antibody titers of PPV in NPF were observably higher than those in PF groups at high dose (*P* < 0.05). On days 14 and 21 after challenge, HI antibody titers of PPV in NPF were markedly higher than those in PF groups at high and middle dose (*P* < 0.05).

#### 3.2.4. The Variation of Serum IL-2, IL-6, and *γ*-IFN Content

**Figure 6:**
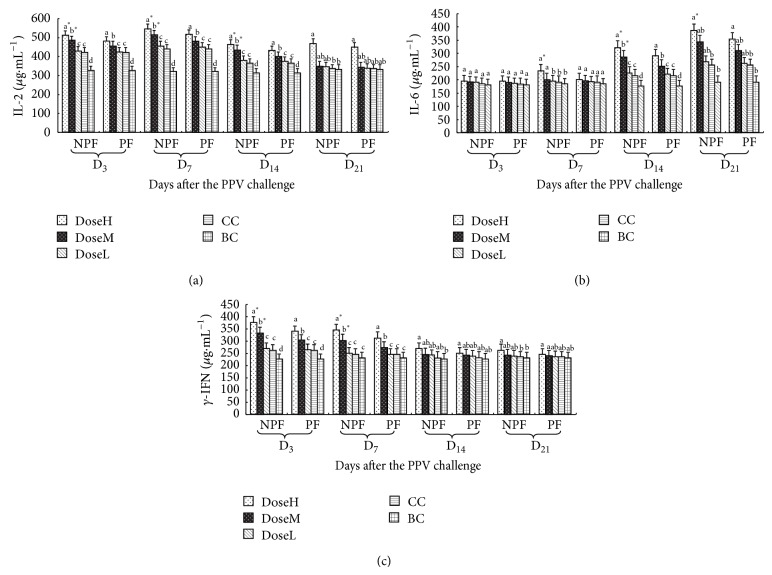
The variation of sera IL-2, IL-6, and *γ*-IFN in every group after challenge (*μ*g·mL^−1^).  ^a–c^Bars in the same mode marked without the same superscripts differ significantly and  ^*^ in the same time differ significantly between NPF and PF (*P* < 0.05). NPF, nanometer propolis flavone; PF, propolis flavone; DoseH, high dose; DoseM, middle dose; DoseL, low dose; CC, challenged control; BC, blank control. (a) The variation of serum IL-2; (b) the variation of serum IL-6; (c) the variation of serum *γ*-IFN.

The variation of IL-2 content of every group in serum is illustrated in Figure 6(a). From 3–14 after challenge, the serum IL-2 content changes of NPF and PF groups were observably higher than those in CC groups at high and middle dose, and on day 21 after challenge, they were significantly higher than those in CC groups at high dose (*P* < 0.05). And on days 3, 7, and 14 after challenge, the serum IL-2 content changes of NPF groups were markedly higher than those in and PF groups at high and middle dose (*P* < 0.05).

The variation of IL-6 content of every group in serum is illustrated in Figure 6(b). From days 7–21 after challenge, the serum IL-6 content changes of NPF groups were observably higher than those in CC groups at high and middle dose (*P* < 0.05). And on day 14 after challenge, the serum IL-6 content changes of PF groups were significantly higher than those in CC groups at high and middle dose, and on day 21 after challenge, they were markedly higher than those in CC groups at high dose (*P* < 0.05). And on days 7 and 21 after challenge, the serum IL-6 content changes of NPF groups were significantly higher than those in and PF groups at high dose, and on day 14 after challenge, they were observably higher than those in and PF groups at high and middle dose (*P* < 0.05).

The variation of *γ*-IFN content of every group in serum is illustrated in Figure 6(c). On days 3 and 7 after challenge, the serum *γ*-IFN content changes of NPF groups were markedly higher than those in CC groups at high and middle dose, and on days 14 and 21 after challenge, they were significantly higher than those in CC groups at high dose (*P* < 0.05). On days 3 and 7 after challenge, the serum *γ*-IFN content changes of PF groups were observably higher than those in CC groups at high and middle dose (*P* < 0.05). And on days 3 and 7 after challenge, the serum *γ*-IFN content changes of NPF groups were significantly higher than those in PF groups at high and middle dose (*P* < 0.05).

#### 3.2.5. The Changes of Weight

**Figure 7:**
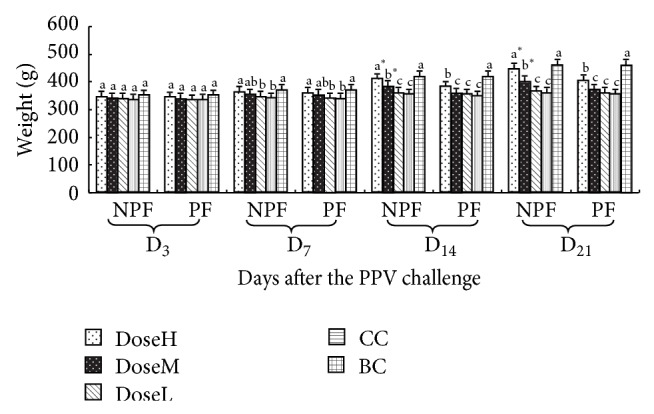
The changes of weight in every group after challenge (g).  ^a–c^Bars in the same mode marked without the same superscripts differ significantly and  ^*^ in the same time differ significantly between NPF and PF (*P* < 0.05). NPF, nanometer propolis flavone; PF, propolis flavone; DoseH, high dose; DoseM, middle dose; DoseL, low dose; CC, challenged control; BC, blank control.

The changes of weight of every group are shown in Figure 7. On day 7 after challenge, the changes of weight in NPF groups were observably higher than those in CC groups at high dose, and on days 14 and 21 after challenge, they were significantly higher than those in CC groups at high and middle dose (*P* < 0.05). From day 7 to 21 after challenged, the changes of weight in PF groups were significantly higher than those in CC groups at high dose (*P* < 0.05). And on days 14 and 21 after challenge, the changes of weight in NPF groups were markedly higher than those in PF groups at high and middle dose (*P* < 0.05).

## 4. Discussion

MTT method is one kind of methods to detect the survival and growth of cells. Its detection principle is that the exogenous MTT could be reduced to water-insoluble violet crystal (Formazan) by the succinate dehydrogenase in living cells and then deposited in cells. But the death cells do not have this function. Dimethyl sulfoxide (DMSO) can dissolve the formazan in cells; the cell absorbance value (*A*_570_ value) could indirectly reflect the number of living cells. Within the scope of a certain number of cells, the content of crystal is proportional to the number of living cells. Therefore, the larger *A*_570_ value is, the more live cells are [16]. Experiment* in vitro* designed three drug-adding patterns, and those are, respectively, corresponding to prevention, sterilization, and treatment of three clinical administrations. In the present experiment, the *A*_570_ values of the NPF at most concentrations were significantly higher than those of PF and virus control groups in three adding modes. These results suggested that NPF could effectively inhibit PPV from infecting PK-15 cell* in vitro*, and the preventative effect was better than those of the sterilization and treatment. The reasons may be that the particle size of NPF is small and quite homogeneous, so it is more advantageous in penetrating cell membrane and being absorbed by cell. When the NPF enters into the organism, it can decelerate the drug release and lengthen the action time of drug in organism by preventing the drug from degrading rapidly. Many other researches* in vivo* also confirmed that the efficacy of drug after nanocrystallization was significantly better than conventional drug [17], which was consistent with this experimental result.

The virus content in infectious cell is also an indicator for antiviral effect of drug [12]. In this test, the content of PPV in PK-15 cell in groups NPF and PF was observably lower than this of the corresponding virus control group in the three modes. In addition, the content of PPV in PK-15 cell group NPF was markedly fewer than this of the corresponding PF group in three adding drug patterns at the proper concentration. Those results indicated that NPF could more effectively inhibit PPV replication on PK-15 cell than that of PF.

In the clinical samples of PPV infectious pigs, PPV was often detected in tissues of lymph nodes, lungs [15, 18]. Guinea pigs is a model animal to discuss the immunity or immune response of PPV vaccine [19]. In this experiment* in vivo*, the PPV content in tissues of lung, gonad, spleen, liver, and blood was measured after challenge. The PPV mainly propagated in gonad, lung, and blood. And at high and middle dose, the PPV content in NPF group was significantly lower than this of the corresponding PF and CC group in tissues of lung, gonad, spleen, and blood. The results suggested that NPF could effectively inhibit PPV infecting tissue of lung and gonad. Gonad is the key organ for PPV replication. NPF could significantly restrain PPV infecting gonad, so NPF was able to effectively alleviate the PPV on reproductive failure and reduce economically the loss in clinical.

The results of* in vivo* test revealed that, during early period of PPV challenge, the HI antibody titer in serum of NPF group was higher than those of PF and CC groups, which indicated that NPF was superior to PF on producing antibody. This could make challenged animals more effectively resist virus infection. Fan et al. [20] also confirmed that PF, designed to microemulsion, was able to significantly increase the thymus and spleen indices and enhance the contents of IgM and IgG in serum. IgM plays an important role in initial stage of anti-infection immunity and is originally produced in the first humoral immune response [21]. IgG possesses high content and long duration and is the primary antibodies for anti-infection immunity [22].

In present experiments, the concentrations of IL-2, IL-6 and *γ*-IFN in serum were determined. The concentrations of IL-2 and *γ*-IFN of NPF group were higher than those of PF and CC groups in early stage and the concentrations of IL-6 of NPF group were higher than those of PF and CC groups in later period at high and middle doses. IL-2 is a cytokines secreted by type 1 helper T cells (Th1 cells) and able to mediate cellular immunity. IL-2 is also able to promote production of immunoglobulins and stimulate the proliferation and differentiation of natural killer cells and is indispensable for the T-cell immunologic memory [23]. IL-6 is secreted by type 2 helper T cells (Th2 cells) and able to coordinate humoral immunity, which is the cytokine most frequently used to detect the antigen specificity of Th2 T cells. *γ*-IFN also is secreted by Th1 cells; it can reflect the basic status of cellular immunity and humoral immunity of organism to certain extent.

The levels of cytokines secretion and antibody titer, respectively, reflected the state of the cellular immunity and humoral immunity in animal organism. Fischer et al. [24] reported that both cellular and humoral immunity played important roles in defense against infectious disease for the host. The present research demonstrated that NPF could improve the cellular and humoral immunity and thus was able to protect guinea pigs from attack by PPV. The ability of modulating the synthesis of antibodies and cytokines secretion is part of the NPF activity compared PF. Despite the fact that the precise action mechanism of NPF on the immune system remains unknown, with further probable amplification of the immune cells reaction can be one of its main antiviral mechanisms [25, 26].

The weight is an important indicator to reflect the situation of organism in disease. Our previous research confirmed that PF or other CHMIs could effectively alleviate the harm caused by virus and increase the weight of challenged animals [12, 27]. The results of the present study also demonstrated that the weights of NPF at high and medium dose groups were significantly higher than those in PF and CC groups in later period, and the weight of NPF at high group was not significantly lower than that in BC group. It indicated that nanotechnology could remarkably enhance the antiviral activity of PF.

## 5. Conclusions

This study assessed the anti-porcine parvovirus activity of NPF and PF* in vitro* and* in vivo*.* In vitro*, NPF could significantly inhibit PPV infecting PK-15 cells compared with PF. And the preventative effect was better than those of the sterilization and treatment.* In vivo*, NPF was able to observably restrain PPV copy in lung, gonad, and blood, lessen the impact of PPV on weight of guinea pigs, and increase hemagglutination inhibition of PPV in serum; it could also improve the contents of IL-2, IL-6, and *γ*-IFN in serum after challenged. Therefore, nanotechnology could observably enhance the anti-PPV activity of PF. Further studies on the safety, stability, and the mechanism of action of NPF are needed to pursue its potential for clinical applications as antiviral drug.

## Figures and Tables

**Table 1:** The maximal safe concentrations of NPF and PF on PK-15 (*A*_570_).

Concentration (*μ*g·mL^−1^)	NPF	PF
2000	0.16 ± 0.02^g^	0.20 ± 0.01^e^
1000	0.21 ± 0.01^f^	0.22 ± 0.02^e^
500	0.30 ± 0.03^e^	0.35 ± 0.01^d^
250	0.56 ± 0.01^a^	0.49 ± 0.02^a^
125	0.46 ± 0.02^b^	0.45 ± 0.02^b^
62.5	0.43 ± 0.01^bc^	0.42 ± 0.01^bc^
31.2	0.41 ± 0.03^c^	0.39 ± 0.02^c^
15.6	0.38 ± 0.01^cd^	0.37 ± 0.03^cd^
7.8	0.36 ± 0.03^d^	0.35 ± 0.01^d^
3.9	0.35 ± 0.02^d^	0.35 ± 0.02^d^
Cell control	0.35 ± 0.01^d^	0.34 ± 0.02^d^

^a–g^Data within a column without the same superscripts differ significantly (*P* < 0.05).

NPF, nanometer propolis flavone; PF, propolis flavone.

## Abbreviations

PPV: Porcine parvovirus
NPF: Nanometer propolis flavone
PK: porcine kidney
PF: Propolis flavone
HI: Hemagglutination inhibition
PCV: Porcine circovirus
CHMs: Chinese herbal medicines
MTT: 3-(4,5-Dimethylthiazol-2-yl)-2,5-diphenyltetrazolium bromide
RTFQ: Real-time fluorescence quantitative
PBS: Phosphate buffer solution
FBS: Fetal bovine serum
DMSO: Dimethyl sulfoxide
CPE: Cytopathic effect
CC: Challenge control
BC: Blank control
IL: Interleukin
IFN: Interferon
ELISA: Enzyme-linked immunosorbent assay.
